# Arabic validation of the Reproductive Autonomy Scale among Egyptian women

**DOI:** 10.1186/s42506-026-00215-4

**Published:** 2026-04-20

**Authors:** Marwa A. Orabi, Omaima El-Gibaly, Doaa M. Osman

**Affiliations:** 1 Demography and Reproductive Health, Public Health and Community Medicine Department, Faculty of Medicine, Assiut University, Assiut City, Egypt; 2 Public Health and Community Medicine Department, Faculty of Medicine, Assiut University, Assiut City, Egypt

**Keywords:** Reproductive Autonomy Scale, Egypt, Validation, Women

## Abstract

**Background:**

The Reproductive Autonomy Scale (RAS) is a recent English validated reliable tool for measuring reproductive autonomy created by Ushma Upadhyay in the United States. It assesses women’s control over decisions related to contraception, pregnancy, and childbearing. RAS has not yet been validated for Arabic-speaking populations. The current study aimed to validate the Arabic version of the RAS among Egyptian married women in the reproductive age group.

**Subjects and methods:**

A cross-sectional study was conducted in three randomly selected primary health care centers among 400 Egyptian married women. Data was collected using an interview-administered questionnaire that included personal characteristics, women autonomy scale, and RAS. A standard forward-backward translation of RAS was done. The reliability of the translated scale was tested by Test- retest reliability and evaluation of the intra-class correlation coefficient (ICC). Convergent validity was assessed by correlating the RAS with the women’s autonomy scale. Confirmatory and exploratory factor analyses (CFA & EFA) were used to test construct validity.

**Results:**

The final Arabic validated form had excellent repeatability levels among the studied Egyptian women (ICC = 0.912). EFA of the long form of RAS resulted in 11 items captured by three factors: freedom from coercion, communication, and decision-making. Total variance explained was 60.37%. The alpha reliability coefficient for the entire scale was 0.74. CFA showed that all items loaded significantly on their hypothesized three latent factors. The fit indices were acceptable (TLI = 0.97; CFI = 0.98; RMSEA = 0.04 and SRMR = 0.04).

**Conclusions:**

The final Arabic validated form has excellent repeatability levels and good face validity.

**Supplementary Information:**

The online version contains supplementary material available at 10.1186/s42506-026-00215-4.

##  Introduction

 Reproductive autonomy refers to a woman’s ability to make decisions regarding when and whether to have children. It includes the power to decide on contraception use, pregnancy, and childbirth without coercion or external interference [1, 2]. Reproductive autonomy is a fundamental reproductive right and is crucial for women’s well-being, though it often remains a low priority in many societies [1].

Women’s reproductive autonomy offers numerous benefits for both women and society [1–3]. It is crucial for promoting women’s rights, health, empowerment, and equality. It has far-reaching benefits that extend to individuals, families, communities, and societies, leading to improved well-being and progress [4–6].

The Reproductive Autonomy Scale (RAS) is a validated tool for measuring reproductive autonomy created by Ushma et al. in the United States in 2014. It assesses a woman’s control over decisions related to contraception, pregnancy, and childbearing. This scale was initially developed with 26 items, and later reduced through factor analysis to 14 items. Those 14 items are grouped into three subscales: freedom from coercion, communication, and decision-making [2].

It is considered reliable for evaluating reproductive autonomy and assessing interventions aimed at enhancing reproductive autonomy, such as family planning programs, and for screening clients at risk of reproductive coercion [2, 7]. It can be applied in reproductive health programs both in the U.S. and globally.

The RAS is available in English, Spanish, and Portuguese. A Vietnamese study validated the scale with moderate to high internal consistency, but suggested the need for adaptations for populations outside the U.S [8].

To date, no studies have yet validated the scale for Arabic-speaking populations. This study aimed to support reproductive health by developing a validated Arabic measurement tool specific to the Egyptian context.

## Methods

### Study design and site

A cross-sectional study design was applied. The study was conducted in three randomly selected primary health care centers (PHCs) at Assiut district, Assiut Governorate, in Upper Egypt. Two urban PHCs and one rural PHC.

### Study population and sampling

The study population comprised currently married women in the reproductive age group (15–49 years) who attended primary health care centers for services (vaccination of their children, family planning services, and medical care for their children). The study excluded current pregnant women, breastfeeding women (until 6 months), women with the intention to get pregnant within the next 12 months, and those whose husbands are not living with them (e.g., traveling abroad).

### Sample technique and size

A purposive non-probability sampling technique was applied for the recruitment of the studied women.

For calculating sample size in validation studies, the guidelines recommend applying the respondent-to-item ratio. The ratio ranges from 5:1, 10:1, 15:1 or 30:1 [9].

The current study tested the validation for the long form of RAS, which has 26 items. Based on the previously mentioned guide, the study applied the rule of 15 respondents for each variable, with a total of 390 respondents (15 respondents *26 variables). The sample was increased to 400 to compensate for any dropout or refusal.

A total of 450 women were invited to participate while awaiting consultations at the PHC centers. Of these, 15 declined (primarily citing limited time), and 35 were excluded for not meeting the inclusion criteria. A final sample of 400 was enrolled, yielding a response rate of 400/ 415 = 96.4%.

### Data collection and study tool

Data were collected through a semi-structured interviewer-administered questionnaire. It lasted four months from August 2022 to November 2022. For each case, the questionnaire filling took 20–30 min on average. After completion of the questionnaires and during collection, a review of the questionnaires was conducted to ensure the absence of any missing items. The questionnaire included:

1. *Sociodemographic characteristics*: Age, residence, family type, education, and occupation of the participating women.
2. *Obstetric*,* reproductive*,* and contraceptive history*: It included parity, history of abortion or stillbirth, number and type of living children (females or males), current use of contraceptives (yes, no), and nature of the used method (modern or traditional).
3. *Women’s autonomy*: It includes four measures: movement autonomy, financial autonomy, household decision-making, and domestic violence attitude. Standardized scoring for each domain of the women’s autonomy scale was performed according to the scale guide [10]. Seventy-six women (those without children or those whose children were less than 5 years old) were excluded from calculating household decision-making scores and total women’s autonomy score, as they were not applicable for answering questions related to parenting.
  a) Mobility autonomy: Assesses whether women need permission to visit places like markets, health centers, or relatives' homes. Higher scores indicate more freedom in movement decisions.
  b) Financial autonomy: This is a binary measure, based on whether women have access to household money and personal savings, land, or valuables. A "yes" answer to either of these indicates financial autonomy.
  c) Household decision-making: Measures who have control over various decisions at home, such as purchases, food, medical care, and child-related decisions. Two variables are created:
    • Individual household decision-making: It counts how often the respondent makes decisions independently.
    • Joint household decision-making: It counts decisions made jointly with others in the household.

Higher counts indicate more involvement in household decisions.

- d) Domestic violence attitudes: A scale based on seven questions assessing whether a husband is justified in beating his wife for specific reasons, such as neglecting children or refusing sex. Higher scores indicate stronger acceptance of domestic violence.
- 4. *Reproductive autonomy scale*: The study used the long form of RAS. It is composed of 26 items [2]. (see supplementary file 1)

### Steps of translation and validation of the questionnaires

1. *Translation and cross-cultural adaptation* of RAS from English into Arabic language: the procedures for translating the scale were based on the guidelines for translation and cross-cultural adaptation [11, 12].
  - Cultural adaptation: since the RAS scale was originally in English, it underwent cultural evaluation to ensure its relevance and effectiveness for the Egyptian (Arabic-speaking) population. Eight experts conducted this cultural adaptation. Forward translation: Two independent persons translated the RAS from English into Arabic at the same time. The first was a public health physician (A reproductive health specialist), informed about the study’s purpose and goals, and the second was a professional translator in the faculty of literature, Assiut University, without any medical background.
  - Expert committee review (synthesis): The team of expert committee consist of the three authors of the study, one professor at the faculty of education who is expert in women autonomy research, one professor at public health who is expert in reproductive research, and three reproductive health specialists working in the Egyptian Ministry of Health and experienced in communicating with the women in the reproductive health services at the Egyptian community. The differences between the two translations were discussed and resolved, and a consensus was developed about the Arabic wording of each item.
  - Backward translation: Two backward translations were carried out by two independent bilingual experts, who were blinded to the original English version.
  -  Expert committee review: the team discussed the discrepancies that were found between the original questionnaire and the back-translated versions and developed an Arabic version for cognitive testing.
  -  Cognitive testing of the translated version was done by open interviews with 10 women using the final translated version and asking them after each question if they understood what the question was assessing. The participants’ suggestions were evaluated by the research team and were added, keeping the same concepts, to be more understandable.

A final Arabic version was produced and tested by another 5 women to ensure it was completely understandable.

2. *Testing the reliability of the translated version*:

Test-retest reliability refers to the extent to which individuals’ responses to the questionnaire items remain relatively consistent across repeated administrations of the same questionnaire. Test-retest reliability can be evaluated using the intra-class correlation coefficient (ICC) [13].

It is suggested that ICC values less than 0.5 are indicative of poor reliability, values between 0.5 and 0.75 indicate moderate reliability, values between 0.75 and 0.9 indicate good reliability, and values greater than 0.90 indicate excellent reliability [14].

To measure the reliability of RAS, a pilot study was conducted on a sample of 10% of the estimated sample size (40 participants). The interviews were repeated twice with 40 women two weeks apart by the same researcher. The data of the pilot study, which had been collected from those 40 women, were restricted to testing the reliability and were not included in the validation analysis (400 women).

- 3. *Validating the translated RAS*:
- a) Face validity: It is about whether a test appears to measure what it is supposed to measure. This type of validity is concerned with whether a measure seems relevant and appropriate for what it’s assessing on the surface.

To assess face validity, we asked two groups of people. The first group includes three experts who have a deep understanding of research methods. The second group includes four potential participants (married women in the reproductive age group) to provide us with valuable insights we may otherwise miss. The researchers asked them the following question: Are the components of the RAS (e.g., questions) relevant to the concepts of reproductive autonomy?

b) Convergent validity: The RAS was a novel questionnaire that measured new aspects of women’s autonomy. The researchers substituted the absence of similar Arabic validated scales measuring these new aspects by correlating the tested questionnaires with a scale measuring other aspects of women’s autonomy.
c) Construct validity:

- 1- Exploratory factor analysis (EFA):

To assess whether the sample is adequate for factor analysis, the anti-image matrix was examined. It confirmed with the desirable cutoff points; the diagonals were greater than 0.5, a KMO value of 0.70 or higher, and a significant Bartlett’s test [15].

Factorability was examined by producing a correlation matrix and the presence of correlation coefficients between items greater than 0.3 [16].

The cutoff for rotated factor loading onto a primary factor was set at 0.40 and the cross-load of absolute value was not allowed to exceed 0.3 on two or more factors [17]. Only the factors with a minimum of three items were retained [18]. 

Principal components analysis with oblique (Promax) rotation was used to allow for the naturally occurring correlation between factors. The number of factors to retain within each model was determined after examining the scree plot [19], maintaining the number of factors with eigenvalues greater than 1 [20], and interpreting each factor [21]. These criteria ensure that the retained items were informative and discriminated between factors.

- 2- Confirmatory factor analysis (CFA):

The factor structure obtained via EFA was subjected to CFA with maximum likelihood (ML) estimation. The model fit was evaluated by goodness of fit (GFI), the comparative fit index (CFI), Tucker-Lewis index (TLI), the root mean square error of approximation (RMSEA), and the standardized root mean squared residual (SRMR).

- Values for GFI, TLI, and CFI indices > 0.95 indicate very good fit [22]. Values 0.90 or above are considered evidence of acceptable fit [23].
- RMSEA value of < 0.08 is considered acceptable [24, 25].
- SRMR values up to 0.05 are considered indicative of a close-fitting model. Values between 0.05 and 0.10 suggest an acceptable fit [23].

Cronbach’s alpha coefficients were used to assess the internal consistency of the scale; an alpha level of 0.70 is considered an acceptable level of self-consistency [26], and values between 0.08 and 0.90 were considered very good [16].

### Statistical analysis

Data were entered and analyzed using the Statistical Package for the Social Sciences software (SPSS) version 20, SPSS version 27, and Stata, IC 16.

Descriptive statistics, reliability tests, correlation analyses, and exploratory factor analysis (EFA) were conducted with SPSS. Confirmatory factor analysis (CFA) was performed using Stata IC 16.

## Results

### Section 1: descriptive statistics

Table 1 shows the socio-demographic characteristics and reproductive history of the studied women. The age of the studied women ranged from 19 to 49 years, with a mean age of 30.63 ± 6.13 years. A significant proportion of the participants (75.8%) resided in urban areas, and 80% lived in nuclear families. In terms of women’s education, 25.5% had university or post-university education, while 14.5% were illiterate or could only read and write. Most women (84%) were housewives.

Regarding reproductive history, half of the women had two or three childbirths. Nearly 64% had children of both sexes, and one-third (32.8%) reported a history of abortion, while a small percentage (4.8%) experienced a stillbirth. The majority (95%) were using a contraceptive method, and modern contraceptive methods were the most commonly used method (95%).

**Table 1:** Demographic characteristics and reproductive history of studied Egyptian women

Variable	N= 400 Frequency (%)
Wife's age in years: Mean ± SD (Range)	30.63± 6.13 (19 – 49)
Family type	
Nuclear	320 (80%)
Extended	80 (20%)
Residence	
Rural	101(25.3%)
Urban	299 (74.7%)
Wife's education categories	
Illiterate / Read & write	58 (14.5%)
Primary / Preparatory	81 (20.3%)
Secondary / Intermediate education	159 (39.7%)
University / Post-university	102 (25.5%)
Wife occupation categories	
Housewife	336 (84%)
Working	64 (16%)
Parity	
One	53 (13.3%)
2-3	222 (55.5%)
4 or more	105 (31.2%)
Number of living children	
One child	52 (13%)
2-3 children	229 (57.3%)
4 children or more	119 (29.7%)
Sex of living children	
Males	72 (18%)
Females	73 (18.3%)
Both	255 (63.7%)
History of abortion	
Yes	131 (32.7%)
No	269 (67.3%)
History of stillbirth	
Yes	19 (4.8%)
No	381 (95.2%)
Usage of contraceptive	
Yes	380 (95%)
No	20 (5%)
Type of the method used (n=380)	
Modern methods	362 (95.3%)
Traditional methods	18 (4.7%)

Table 2 displays the domains of the women's autonomy scale among the studied women. Women who have financial autonomy formed more than half of the studied women (53%). The total women's autonomy score ranged from 5 to 32, with a mean value of 21.2 ± 4.4. 

**Table 2:** Women's autonomy domains among studied Egyptian women

Variable	Total (N=400)
Decision-making domain (n=324)^∞^	
Individual decision-making	4.08 ± 2.48 (0-9)
Joint decision-making	3.03 ± 2.34 (0-9)
Mobility domain	8.89 ± 2.33 (4-16)
Domestic Violence domain	4.72 ± 1.88 (0-7)
Financial autonomy	
Not having financial autonomy	188 (47%)
Having financial autonomy	212 (53%)
Total women autonomy score ∞ (n=324)	21.2 ± 4.4 (5-32)

Data were presented in the form of mean ± SD (range) or frequencies and percentages

^∞^Seventy-six women were excluded (those without children or those whose children were less than 5 years old)

Descriptive analysis of the long form of the reproductive autonomy scale among the studied Egyptian women. (see supplementary file 2)

### Section (2): Validation of the RAS

A- Translation and cultural adaptation.

B- Repeatability testing of the reproductive autonomy scale among a pilot sample.

C- Face validity

D- Convergent validity (Correlation of reproductive autonomy scale with other related scales)

E- Exploratory factor analysis of reproductive autonomy scale

F- Confirmatory factor analysis of reproductive autonomy scale

- A. *Translation and cultural adaptation*
  * English to Arabic forward translation:- Almost all phrases of the English RAS questionnaire were easily translated into Arabic. However, some words and phrases were not suitable for the cultural context of the Egyptian people. So, these words were replaced with other culturally accepted words without changing the conceptual meaning of the items. Specifically, the phrase "seek adoptive parents" in item 4 was replaced by "seeking help from someone in your family to raise the child". The word "partner" was replaced by "husband". The word "sex" was replaced by "sexual marital relation", and the phrase "who has the most say" in items 1, 2, 3, and 5 was replaced by "who has the final say" to be more understandable.
  * Arabic to English back translation: Back translations were very similar to the original questionnaire except for the replaced words, while preserving the main conceptual meanings of every sentence. The minor discrepancies in translation were identified, discussed, and a consensus Arabic version emerged after discussion by the research committee.
  * Cognitive testing: after interviewing 10 women in the reproductive age group to assess their level of understanding of all the translated questionnaire phrases’ meanings, it was found that, some questions in the RAS which ask about "who has the final say" need some clarification so the phrase "If there was a difference of opinion between you and your husband" was added. After making the required modifications in the wording of the translated questionnaire and developing a final Arabic version, it was tested by another 5 women, and was found to be simple and easily understood by women.
- B. Test- retest reliability (Repeatability) of reproductive autonomy scale among a pilot sample of women (n=40). Both the long form and the final validated form had excellent repeatability levels among the pilot sample of the studied Egyptian women in the reproductive age group. The intra-class correlation coefficients for the final validated form and its domains (decision making, communication, freedom from coercion) were 0.912, 0.823, 0.905, and 0.865, respectively (Table 3).
- C.* Face validity*: after asking the group of experts and the group of participants about the relevance of items of RAS to the concept of reproductive autonomy, they reported that the items were relevant to the concept of reproductive autonomy. So, the scale has good face validity.
- D. *Convergent validity *was assessed by conducting a correlation between the reproductive autonomy scale and the women's autonomy scale and its domains. The validated form of the reproductive autonomy scale was significantly positively correlated with all domains of the women's autonomy scale (P < 0.05). The correlations range from r = 0.114 (Individual Decision-making) to r = 0.308 (Violence domain). According to Cohen’s criteria, these represent small-to-medium effect sizes [27]. While these relationships are statistically significant, the low r values indicate that general women's autonomy explains only a small fraction of the variance in reproductive autonomy. These results characterize RAS and general autonomy as related but distinct constructs. In psychometric validation, high convergent validity (e.g., r > 0.50) is expected only when scales measure the same underlying trait. However, "Reproductive Autonomy" is a domain-specific construct. It suggests that reproductive autonomy is a distinct construct that is not fully captured by traditional measures of women’s empowerment in Egypt (Table 4).
- E. * Construct validity of the RAS*:

1. Exploratory Factor Analysis (EFA) of the RAS scale: (see full output of EFA in supplementary file 4).

**Table 3:** Repeatability of RAS among a pilot sample of Egyptian women (*n*=40)

Variable	ICC- 95%CI	Cronbach's Alpha	P value
Long form of the RA scale	0.916 (0.841 - 0.956)	0.916	< 0.001
Final RA scale	0.912 (0.834 - 0.953)	0.912	< 0.001
Decision-making subdomain	0.823 (0.666 - 0.907)	0.823	< 0.001
Communication subdomain	0.905 (0.821 - 0.950)	0.905	< 0.001
Coercion subdomain	0.865 (0.744 - 0.928)	0.865	< 0.001

ICC (intra-class correlation), 95% CI: 95% confidence interval

**Table 4:** Correlation between the long form of RAS and the women's autonomy scale and its domains among the studied Egyptian women (*n*=400)

Domains of women's autonomy scale	RAS	
	Correlation Coefficient	P value
Individual decision-making domain^∞^ (n= 324)	0.114	(0.040)
Joint decision-making domain^∞^ (n= 324)	0.164	0.003
Mobility domain	0.258	< 0.001
Violence domain	0.308	< 0.001
Financial domain©	0.304	< 0.001

Pearson correlation

©Spearman's correlation

^∞^ Seventy-six women without children and those whose children were less than 5 years old were excluded.

**Fig. 1:**
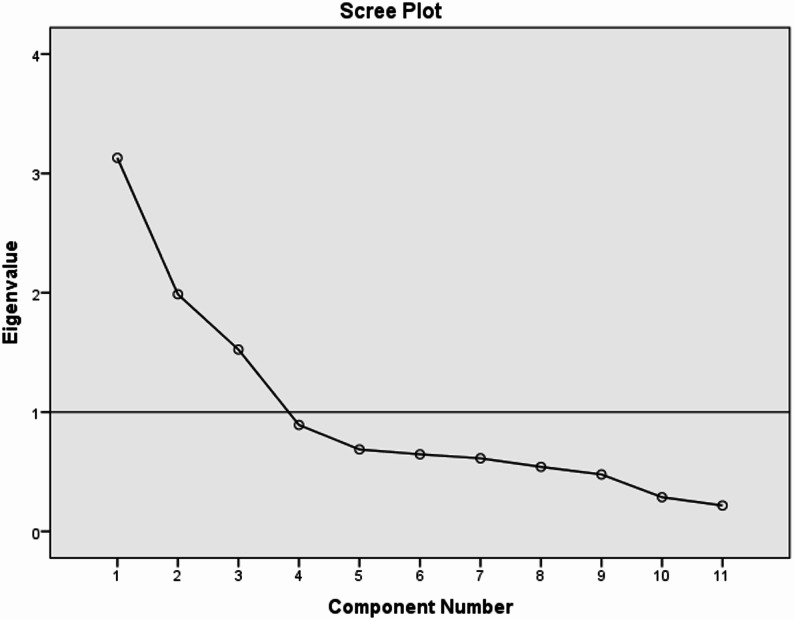
Scree plot of exploratory factor analysis of the reproductive autonomy scale among the studied Egyptian women

The results of EFA revealed that the determinant of the matrix was not zero, the KMO measure of sampling adequacy was 0.719, and Bartlett’s test of sphericity was significant (*p* < 0.001). A three-factor solution was obtained, which explained 60.37% of the variance as demonstrated by the scree plot (Fig. 1).

In the first round of EFA, the 26 items were distributed into 9 factors. After applying the criteria for retaining items and factors, the following items and factors were deleted. Items 7, 11, and 26 were deleted because they were cross-loaded on more than one factor by > 0.3. Factors 4, 5, 6, 7, 8, and 9 were deleted as each one of these factors contains < 2 items. So, items 5, 8, 12, 13, 14, 15, 16, 20, 23, 24, and 25 were deleted. Next, we ran EFA for the second round and ended up with three factors and 12 items. Item 6 (My husband would support me if I wanted to use a method to prevent pregnancy) was deleted after consensus of the research committee, as it doesn’t match the general concept of its factor (freedom from coercion).

Table 5 shows the final version of the RA scale, consisting of 11 items that explained 60.37% of the variance. The alpha reliability coefficient for the entire scale was 0.74. All the items loaded on their designated constructs, which ranged from 0.74 to 0.89 (Freedom from coercion), 0.73 to 0.87 (Communication), and 0.45 to 0.79 (Decision-making).

**Table 5:** The eleven constitutive items and their corresponding factor loadings across the three subscales of the Reproductive Autonomy Scale

Subscale	Item	Rotated factor loading^∞^
Freedom from coercion ^π^		
Alpha = 0.822Mean = 14.317Range = 4 – 16	1. My husband has stopped me from using a method to prevent pregnancy when I wanted to use one. (Q18)	0.898
	2. My husband has messed with or made it difficult to use a method to prevent pregnancy when I wanted to use one. (Q19)	0.830
	3. If I wanted to use a method to prevent pregnancy, my husband would stop me. (Q21)	0.756
	4. My husband has pressured me to become pregnant. (Q22)	0.734
Communication		
Alpha = 0.737Mean = 8.705Range = 3 - 12	1. It is easy to talk about sexual marital relations with my husband. (Q9)	0.725
	2. If I didn’t want to have a sexual marital relationship, I could tell my husband. (Q10)	0.870
	3. A woman can refuse sexual marital relations with her husband for any reason. (Q17)	0.856
Decision-making^£^		
Alpha = 0.646Mean = 8.960Range = 4 – 12	1. Who has the final say about whether you use a method to prevent pregnancy? (Q1)	0.794
	2. Who has the final say about which method you would use to prevent pregnancy? (Q2)	0.450
	3. Who has the final say about when you have a baby in your life? (Q3)	0.773
	4. If you became pregnant but it was unplanned, who would have the most say about whether you would raise the child, seek help from someone in your family to raise the child, or have an abortion? (Q4)	0.712
Full Scale		
Alpha = 0.736Explained variance: 60.375		

∞ Rotated factor loading represents how the item is weighed for each factor and the correlation between the item and the factor

π £Response choices were strongly agree, agree, disagree, strongly disagree

£ Response choices were my partner or someone else, me and my partner (or someone else) equally, or me

The freedom from coercion subscale consisted of four items [18, 19, 21], and [22] that explained 28.46% of the variance, and Cronbach’s alpha was 0.82. The communication subscale consisted of three items [9, 10], and [17] that explained 18.06% of the variance, and the Cronbach’s alpha was 0.74. The decision-making subscale consisted of four items (1, 2, 3, and 4) that explained 13.85% of the variance with a Cronbach’s alpha of 0.65 and McDonald’s omega of 0.69.

**Fig. 2:**
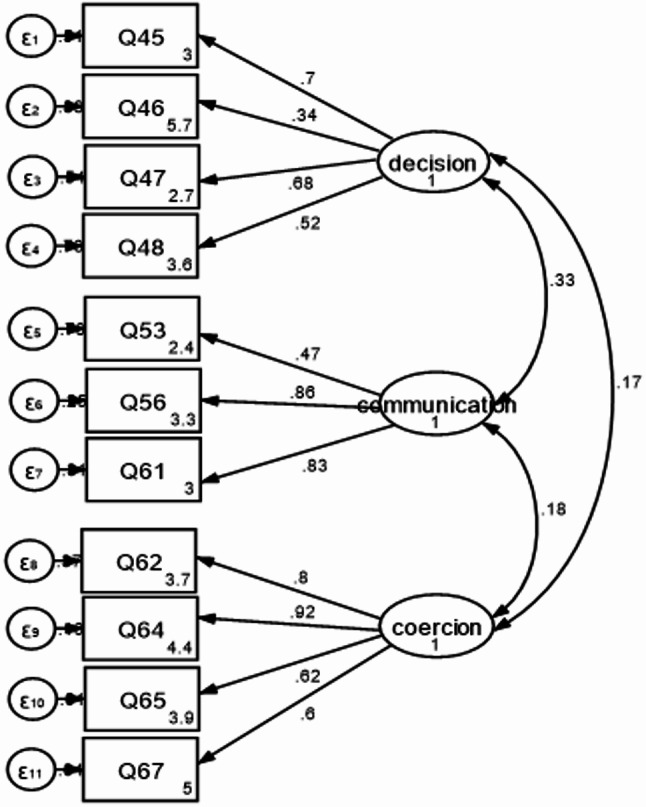
Confirmatory factor analysis of reproductive autonomy scale among studied women, Assiut District

- 2. *Confirmatory Factor Analysis (CFA) of RAS*:

CFA was performed to confirm the three-factor structure model obtained earlier through EFA. All items loaded significantly on their hypothesized three latent factors. The fit indices were acceptable (TLI = 0.97; CFI = 0.98; RMSEA = 0.04 and SRMR = 0.04). As shown in Fig. 2, all factor loadings were significant (*p* < 0.05) and ranged from 0.34 to 0.7 (Decision-making), 0.47 to 0.68 (Communication), and 0.6 to 0.92 (Freedom from coercion). There was a positive correlation between the three latent factors; the strongest correlation was found between the communication and the decision-making latent factors.

Description of the final validated Arabic form of the reproductive autonomy scale among the studied Egyptian women (see supplementary file 3).

## Discussion

The present study was a cross-sectional study conducted among 400 married women at primary health care centers, Assiut Governorate, Upper Egypt. It aimed to validate an Arabic version of the RAS within the Egyptian context. The key findings included.

The socio-demographic characteristics of our sample of women, in terms of education and employment status, are more or less similar to population representative samples of the Egypt Family Health Survey (EFHS ) 2021 or census office estimates of Egypt (CAPMAS) [28, 29].

However, concerning contraceptive use, the study subjects had a higher contraceptive usage (95%) compared to the EFHS survey 2021, which reported a contraceptive usage rate of 66.4% among currently married women 15–49 Years of age [28]. This high rate may be due to a difference in the PHC services users from the general population, and coming mainly for vaccination of their children, and are more likely to be using family planning methods after having a child.

Concerning the validation of the RAS, in this study, the researchers examined the structure, validity, and reliability of the RA scale using a sample of Egyptian women in the reproductive age group. The original long form of RAS was developed in the United States in 2014 by Ushma et al. It consisted of 26 items and was reduced to 14 items distributed over three subscales that included decision making, communication, and freedom from coercion [2].

Similarly, the EFA in the Arabic version of the RAS in the current study resulted in a reduction of the 26 items into three factors (constructs) named as follow decision making, communication, and freedom from coercion. However, it retained only 11 items (Cronbach’s alpha = 0.74), which explained 60.38% of the variance, and were distributed over three constructs.

Specifically, when we compare the results of the current study and the results of the developer of the RA scale, we found that the items retained in the decision-making factor are the same items retained by the developer.

Regarding the communication factor, the final Arabic validated scale includes three items as follows: ‘It is easy to talk about sex with my husband’, ‘if I didn’t want to have sex, I could tell my husband’, and ‘a woman can refuse sex with her husband for any reason’. But the final validated short version of the English scale contains five items. The first two items are included in both the final validated short version English scale and the final Arabic validated scale, but the third item is included only in the final validated Arabic scale. In addition, the American short scale contains three more items; ‘My partner would support me if I wanted to use a method to prevent pregnancy’, ‘if I was worried about being pregnant or not being pregnant, I could talk to my partner about it’ and; if I really did not want to become pregnant, I could get my partner to agree with me’.

It was noticed that all items that were retained under the communication factor in our study addressed issues related to sexual marital relations. Moreover, the item (My husband would support me if I wanted to use a method to prevent pregnancy) was loaded on the freedom from coercion factor, so it was deleted. The item ‘if I was worried about being pregnant or not being pregnant, I could talk to my husband about it’ does not match our cultural context because all the studied women were married (in the Egyptian and Arabic culture, the relation is always within the marriage context, not a partnership relation like European and American societies). Therefore, it is normal for Egyptian women to tell their husbands about their pregnancy without being afraid from rejecting the pregnancy or avoiding bearing the child responsibility.

Regarding freedom from coercion factor, the final Arabic validated scale includes the following four items; ‘my husband has stopped me from using a method to prevent pregnancy when I wanted to use one’, ‘my husband has messed with or made it difficult to use a method to prevent pregnancy when I wanted to use one’, ‘if I wanted to use a method to prevent pregnancy my husband would stop me’. ‘My husband has pressured me to become pregnant.

The final validated American scale includes the same four items plus one additional item, which is ‘my husband has made me use a method to prevent pregnancy when I did not want to use one’. This item was not included in the Arabic scale. Maybe because the majority of studied cases reported that their husbands wished to have a child and didn’t want their wives to use a method.

We also conducted CFA using structural equation modeling. The model fit from the CFA provided further evidence that RA encompassed three latent factors, as theorized by the developers of the RAS. The convergent validity of the RAS was demonstrated through its positive correlation with all domains of the Women’s Autonomy Scale. The Arabic version of the RAS also demonstrated excellent pre-post reliability levels (The intra-class correlation coefficients for the final validated form and its domains (decision making – communication – freedom from coercion) were 0.912, 0.823, 0.905, 0.865, respectively).

###  Study limitations

While there were multiple strengths of the current study, there were still some limitations that should be considered when interpreting our findings.

First: in the current study, we used purposive, non-probability sampling of married women attending three PHC centers in Assiut district. PHC attendees are more likely to be currently using modern contraception, which is reflected in the very high contraceptive prevalence in our sample (95%) compared with the national average of about 66% (Egypt FHS,2021). As a result, the sample has limitations in representing all married women in Assiut or Egypt, particularly those who do not attend PHC facilities, have limited access to services, or are a large number of non-contraception users. This selection bias may limit the generalizability of prevalence/ estimates of reproductive health indicators. However, our primary objective was to evaluate the psychometric properties (reliability, factor structure, and construct validity) of the Arabic Reproductive Autonomy Scale. Psychometric validation does not strictly require a probability sample [30]. We strongly recommend that future studies evaluate the scale in more diverse and representative population-based samples.

Second, the RAS is a novel questionnaire that measures new aspects of women’s autonomy. The researchers substituted the absence of similar Arabic validated tools measuring these new aspects by correlating the tested questionnaire with a scale measuring women’s autonomy.

Third, the decision-making subscale consists of four items with factor loadings between 0.45 and 0.79. Cronbach’s alpha was 0.65, which is slightly below conventional rules-of-thumb (i.e., 0.70), but it must be interpreted considering the subscale’s brevity and item characteristics. Alpha is sensitive to the number of items and tends to be lower for short scales, even when items load adequately on a common factor [31]. In our data, three items showed strong factor loadings (> 0.70), whereas one item (choice of contraceptive method) had a more modest loading (0.45), which likely reduced alpha. Also, it is consistent with other international validations of the RAS, where the Decision-making subscale often yields lower reliability than “Coercion” or “Communication.”

Moreover, we have calculated McDonald’s omega, which was 0.69, indicating acceptable internal consistency for a very brief scale. Alpha is known to be sensitive to the number of items and to underestimate reliability when items are congeneric rather than strictly tau-equivalent [31]. We therefore regard this reliability estimate as borderline but acceptable for group-level research, while recommending further refinement of this subscale in future work. We have chosen to retain this subscale to maintain the theoretical integrity of the original instrument [2] as the decision-making is an important part of the conceptual framework during the development of the reproductive autonomy scale, and to allow for cross-cultural comparisons.

## Conclusions

The study resulted in an Arabic valid and reliable short version of RAS, which consists of eleven elements in three domains (decision making, communication, and freedom from coercion), that can be used to support family planning and reproductive health programs.

## Supplementary Information

Supplementary Material 1: Supplementary file 1: It is a Word file containing the questions of the long form of RAS.

Supplementary Material 2: Supplementary file 2: It is a Word file containing a description of the long form of RAS.

Supplementary Material 3: Supplementary file 3: It is a Word file containing a description of the final validated Arabic version of the RAS.

Supplementary Material 4: Supplementary file 4: It is an SPSS file containing the full output of the EFA.

## Data Availability

The datasets generated and analyzed during the current study are available from the corresponding author upon reasonable request.

## Abbreviations

CFA: Confirmatory factor analysis
CFI: The comparative fit index
EDHS: Egypt Demographic and Health Survey
EFA: Exploratory factor analysis
GFI: Goodness of fit
ICC: Intraclass correlation coefficient
RAS: Reproductive Autonomy Scale
RMSEA: The root mean square error of approximation
SRMR: The standardized root mean squared residual
TLI: Tucker-Lewis index
