# Supplementary material for: Arabic validation of the Reproductive Autonomy Scale among Egyptian women
Source: J Egypt Public Health Assoc. 2026 Apr 20;101:12. doi: 10.1186/s42506-026-00215-4 (PMC13096278; doi:10.1186/s42506-026-00215-4)
Supplement: Supplementary file 1 — Supplementary Material 1: Supplementary file 1: It is a Word file containing the questions of the long form of RAS. [file 42506_2026_215_MOESM1_ESM.docx]

Description of final validated Arabic form of reproductive autonomy scale among studied Egyptian women.

| **Variable** | **Freq. (%)** | **Freq. (%)** | | **Freq. (%)** |
| --- | --- | --- | --- | --- |
| **Decision making domain** | **My husband (or someone else^π^)** | **Both me and my husband (or someone else^π^) equally** | | **Me only** |
| 1- Who has the most say about whether you use a method to prevent pregnancy? | 79 (19.8%) | 173 (43.3%) | | 148 (37%) |
| 2- Who has the most say about which method you would use to prevent pregnancy? | 21 (5.3%) | 27 (6.8%) | | 352 (88%) |
| 3- Who has the most say about when you have a baby in your life? | 116 (29%) | 186 (46.5%) | | 98 (24.5%) |
| 4- If you became pregnant but it was unplanned, who would have the most say about whether you would raise the child, seek adoptive parents, or have an abortion? | 60 (15%) | 278 (69.5%) | | 62 (15.5%) |
| **Freedom from coercion** | **Strongly disagree (4)** | **Disagree**  **(3)** | **Agree**  **(2)** | **Strongly agree (1)** |
| 1. My husband has stopped me from using a method to prevent pregnancy when I wanted to use one. | 323 (80.8%) | 40 (10%) | 9 (2.3%) | 28 (7%) |
| 2. My husband has messed with or made it difficult to use a method to prevent pregnancy when I wanted to use one. | 299 (74.8%) | 44 (11%) | 22(5.5%) | 35(8.8%) |
| 3. If I wanted to use a method to prevent pregnancy my husband would stop me. | 279 (69.8%) | 65 (16.3%) | 29 (7.3%) | 27 (6.8%) |
| 4. My husband has pressured me to become pregnant. | 312 (78%) | 59 (14.8%) | 12 (3%) | 17(4.3%) |
| **Communication domain** | **Strongly disagree (1)** | **Disagree**  **(2)** | **Agree**  **(3)** | **Strongly agree (4)** |
| 1. It is easy to talk about sex with my husband. | 97 (24.3%) | 36 (9%) | 144 (36%) | 123 (30.8%) |
| 2. If I didn’t want to have sex, I could tell my husband. | 38 (9.5%) | 42 (10.5%) | 180 (45%) | 140 (35%) |
| 3. A woman can refuse sex with her husband for any reason. | 50 (12.5%) | 62 (15.5%) | 159 (39.8%) | 129 (32.3%) |

**^π^** such as a parent or mother in-law/father in-law
