# Supplementary material for: Arabic validation of the Reproductive Autonomy Scale among Egyptian women
Source: J Egypt Public Health Assoc. 2026 Apr 20;101:12. doi: 10.1186/s42506-026-00215-4 (PMC13096278; doi:10.1186/s42506-026-00215-4)
Supplement: Supplementary file 2 — Supplementary Material 2: Supplementary file 2: It is a Word file containing a description of the long form of RAS. [file 42506_2026_215_MOESM2_ESM.docx]

**Long form of reproductive autonomy (reproductive decision making) among studied women, Assiut District 2023**

| **Variable** | **Freq. (%)** | **Freq. (%)** | **Freq. (%)** |
| --- | --- | --- | --- |
|  | **My husband (or someone else^π^)** | **Both me and my husband (or someone else^π^) equally** | **Me only** |
| Who has the final say about whether you use a method to prevent pregnancy? "If there was a difference of opinion between you" | 79 (19.8%) | 173 (43.3%) | 148 (37%) |
| Who has the final say about which method you would use to prevent pregnancy? "If there was a difference of opinion between you" | 21 (5.3%) | 27 (6.8%) | 352 (88%) |
| Who has the final say about when you have a baby in your life? "If there was a difference of opinion between you" | 116 (29%) | 186 (46.5%) | 98 (24.5%) |
| If you became pregnant but it was unplanned, who would have the most say about whether you would raise the child, seeking help from someone in your families to raise the child, or have an abortion? | 60 (15%) | 278 (69.5%) | 62 (15.5%) |
| Who has the final say about when you have sex? "If there was a difference of opinion between you" | 264 (66%) | 129 (32.3%) | 7 (1.8%) |

**^π^** such as a parent or mother in-law/father in-law

**Long form of reproductive autonomy scale among studied women, Assiut District 2023.Continue.**

| **Variable** | **Strongly disagree (1)** | **Disagree**  **(2)** | **Agree**  **(3)** | **Strongly agree (4)** |
| --- | --- | --- | --- | --- |
| My husband would support me if I wanted to use a method to prevent pregnancy. | 19 (4.8%0 | 12 (3%) | 63 (15.8%) | 306 (76.5%) |
| My husband would support me if I wanted to have a baby. | 73 (18.3%) | 29 (7.3%) | 67 (16.8%) | 231 (57.8%) |
| My husband would support me if I wanted to have an abortion. | 360 (90%) | 11 (2.8%) | 16 (4%) | 13 (3.3%) |
| It is easy to talk about sex with my husband. | 97 (24.3%) | 36 (9%) | 144 (36%) | 123 (30.8%) |
| If I really wanted to have a baby, I could get my husband to agree with me. | 77 (19.3%) | 62 (15.5%) | 129 (32.3%) | 132 (33%) |
| If I really did not want to become pregnant, I could get my husband to agree with me. | 90 (22.5%) | 71 (17.8%) | 124 (31%) | 115 (28.8%) |
| If I didn’t want to have sex, I could tell my husband. | 38 (9.5%) | 42 (10.5%) | 180 (45%) | 140 (35%) |
| If I was worried about being pregnant or not being pregnant, I could talk to my husband about it | 13 (3.3%) | 6 (1.5%) | 105 (26.3%) | 276 (69%) |
| I think it is acceptable for a woman to try to prevent a pregnancy even if her husband wants to have a baby. | 201 (50.3%) | 54 (13.5%) | 67 (16.8%) | 78 (19.5%) |
| I think it is acceptable for a woman to try to get pregnant even if her husband does not want to have a baby | 247 (61.8%) | 71 (17.8%) | 46 (11.5%) | 36 (9%) |
| I think it is acceptable for a woman to have an abortion if she does not want to have a baby | 325 (81.3%) | 32 (8%) | 31 (7.8%) | 12 (3%) |
| A woman can refuse sex with her husband for any reason | 50 (12.5%) | 62 (15.5%) | 159 (39.8%) | 129 (32.3%) |

**Long form of reproductive autonomy scale among studied women, Assiut District 2023.Continue.**

|  | **Strongly disagree (4)** | **Disagree**  **(3)** | **Agree**  **(2)** | **Strongly agree (1)** |
| --- | --- | --- | --- | --- |
| My husband has messed with or made it difficult to use a method to prevent pregnancy when I wanted to use one | 299 (74.8%) | 44 (11%) | 22(5.5%) | 35(8.8%) |
| My husband has made me use a method to prevent pregnancy when I did not want to use one. | 334 (83.5%) | 42 (10.5%) | 13 (3.3%) | 11 (2.8%) |
| My husband has stopped me from using a method to prevent pregnancy when I wanted to use one. | 323 (80.8%) | 40 (10%) | 9 (2.3%) | 28 (7%) |
| If I wanted to use a method to prevent pregnancy my husband would stop me | 279 (69.8%) | 65 (16.3%) | 29 (7.3%) | 27 (6.8%) |
| If I didn’t want to use a method to prevent pregnancy my husband would make me | 283 (70.8%) | 69 (17.3%) | 27 (6.8%) | 21 (5.3%) |
| My husband has pressured me to become pregnant. | 312 (78%) | 59 (14.8%) | 12 (3%) | 17(4.3%) |
| Men need more sex than women do | 8(2%) | 24 (6%) | 43(10.8%) | 325 (81%) |
| Taking care of children is the woman’s job | 18(4.5%) | 57(14.3%) | 57(14.3%) | 268(67%) |
| If a woman gets pregnant, she should have the baby even if she does not want to have a baby | 13(3.3%) | 12(3%) | 54(13.5%) | 321(80.3%) |
| **Reliability of the long form of the RA scale (26 item)** | Cronbach's Alpha = 0.716 | | | |
