# Supplementary material for: Arabic validation of the Reproductive Autonomy Scale among Egyptian women
Source: J Egypt Public Health Assoc. 2026 Apr 20;101:12. doi: 10.1186/s42506-026-00215-4 (PMC13096278; doi:10.1186/s42506-026-00215-4)
Supplement: Supplementary file 3 — Supplementary Material 3: Supplementary file 3: It is a Word file containing a description of the final validated Arabic version of the RAS. [file 42506_2026_215_MOESM3_ESM.docx]

Supplementary files and tables

Supplementary file 1

Long form of reproductive autonomy scale

1. The first five questions ask the respondents to identify the person who has the final say in decision making.
   1. Who has the most say about whether you use a method to prevent pregnancy?
   2. Who has the most say about which method you would use to prevent pregnancy?
   3. Who has the most say about when you have a baby in your life?
   4. If you became pregnant but it was unplanned, who would have the most say about whether you would raise the child, seek adoptive parents, or have an abortion?
   5. Who has the most say about when you have sex?

Each question has three response choices ranged from 1-3 “my husband (or someone else such as a parent or mother in-law /father in-law)= 1, both me and my husband equally (or someone else such as a parent or mother in-law /father in-law)= 2, or only me =3.

1. The rest of the items: Each item has four response choices. For positive items, each statement has 4 responses ranged from 1-4 (“strongly disagree=1, disagree =2, agree=3, strongly agree =4”) as follows:
   1. My partner would support me if I wanted to use a method to prevent pregnancy.
   2. My partner would support me if I wanted to have a baby.
   3. My partner would support me if I wanted to have an abortion.
   4. It is easy to talk about sex with my partner.
   5. If I did not want to have sex, I could tell my partner.
   6. If I were worried about being pregnant or not being pregnant, I could talk to my partner about it.
   7. If I really did not want to become pregnant, I could get my partner to agree with me.
   8. If I really wanted to have a baby, I could get my partner to agree with me.
   9. I think it is acceptable for a woman to try to prevent a pregnancy even if her partner wants to have a baby.
   10. I think it is acceptable for a woman to try to get pregnant even if her partner does not want to have a baby.
   11. I think it is acceptable for a woman to have an abortion if she does not want to have a baby.
   12. A woman can refuse sex with her partner for any reason.

For negative items, respondents asked about their agreement. Answers ranged from 1-4 (“strongly disagree =4, disagree=3, agree=2, strongly agree=1).

- 1. My partner has stopped me from using a method to prevent pregnancy when I wanted to use one?
  2. My partner has messed with or made it difficult to use a method to prevent pregnancy when I wanted to use one.
  3. My partner has made me use a method to prevent pregnancy when I did not want to use one.
  4. If I wanted to use a method to prevent pregnancy my partner would stop me.
  5. My partner has pressured me to become pregnant.
  6. If I didn’t want to use a method to prevent pregnancy my partner would make me.
  7. Men need more sex than women do.
  8. Taking care of children is the woman’s job.
  9. If a woman gets pregnant, she should have the baby even if she does not want to have a baby.

Because all of the negative items are theoretically contrary to reproductive autonomy, we reverse coded the items in this factor to compute the overall alpha score and to calculate a score for each individual. The higher mean scores indicated greater reproductive autonomy of the women.

**Final form of the reproductive autonomy scale:**

1. **Decision making domain**

1- Who has the final say about whether you use a method to prevent pregnancy?

2- Who has the final say about which method you would use to prevent pregnancy?

3- Who has the final say about when you have a baby in your life?

4- If you became pregnant but it was unplanned, who would have the final say about whether you would raise the child, seeking help from someone in your families to raise the child, or have an abortion?

1. **Freedom from coercion**

1. My husband has stopped me from using a method to prevent pregnancy when I wanted to use one.

2. My husband has messed with or made it difficult to use a method to prevent pregnancy when I wanted to use one.

3. If I wanted to use a method to prevent pregnancy my husband would stop me.

4. My husband has pressured me to become pregnant.

1. **Communication domain**

1. It is easy to talk about sexual marital relation with my husband.

2. If I didn’t want to have sexual marital relation I could tell my husband.

3. A woman can refuse sexual marital relation with her husband for any reason.
